# Supplementary material for: The Systems Biology Research Tool: evolvable open-source software
Source: BMC Syst Biol. 2008 Jun 29;2:55. doi: 10.1186/1752-0509-2-55 (PMC2446383; doi:10.1186/1752-0509-2-55)
Supplement: Additional file 1 — SBRT Archive. An archive of the current version of the Systems Biology Research Tool. [file 1752-0509-2-55-S1.zip › sbrt-1.4.0/doc/developers_guide/api/sbrt/kernel/fba/comp/InitialPointComputer.html]

InitialPointComputer


|  |  |  |
| --- | --- | --- |
| **Overview** \| **Package** \| **Class** \| **Use** \| **Tree** \| **Deprecated** \| **Index** \| **Help** | |  |
| **PREV CLASS**   **NEXT CLASS** | **FRAMES**    **NO FRAMES**     **All Classes** |  |
| SUMMARY: NESTED \| FIELD \| CONSTR \| METHOD | DETAIL: FIELD \| CONSTR \| METHOD |  |


---


## sbrt.kernel.fba.comp Class InitialPointComputer

```
java.lang.Object
  sbrt.kernel.fba.comp.InitialPointComputer
```

**All Implemented Interfaces:**: FluxCvComputer

---

``` public class InitialPointComputer extends java.lang.Object implements FluxCvComputer ```

This class is used to compute initial points for coordinate-direction
hit-and-run random flux vector algorithms.

**Author:**
:   This class was written and documented by Jeremiah Wright
    while in the Wagner lab.

---

| **Nested Class Summary** | |
| --- | --- |
| `class` | `InitialPointComputer.Results`             This class is used to represent the results of a |


**Constructor Summary**

`InitialPointComputer(FbaOptComputer computer)`
  
          Constructs a new initial point, or flux vector, computer.


| **Method Summary** | |
| --- | --- |
| `Interval` | `getFluxConstraint(java.lang.String rxnName)`             Returns the current flux constraint on the reaction with the provided name. |
| `java.util.Map<java.lang.String,Interval>` | `getFluxConstraints()`             Returns all current flux constraints. |
| `Fluxome` | `getFluxome()`             Returns the fluxome used by this computer. |
| `double` | `getPIntercept()`             Returns the value of each positioning variable at the edges of the convex polytope. |
| `Interval` | `getRxnExprConstraint(MathExpr<java.lang.String> rxnNameExp)`             Returns the current constraint on the provided expression. |
| `java.util.Map<MathExpr<java.lang.String>,Interval>` | `getRxnExprConstraints()`             Returns all current flux expression constraints. |
| `void` | `restoreFluxConstraint(java.lang.String reactionName)`             Restores the flux constraint for the reaction with the provided name to the value contained in the fluxome. |
| `void` | `restoreFluxConstraints()`             Restores the flux constraints for all reactions in the fluxome to those values contained in the fluxome. |
| `void` | `restoreFluxConstraints(java.util.Collection<java.lang.String> reactionNames)`             Restores the flux constraints for the provided reaction names to the values contained in the fluxome. |
| `void` | `restoreRxnExprConstraint(MathExpr<java.lang.String> expr)`             Restores the constraint on the provided flux expression to the value contained in the fluxome. |
| `void` | `restoreRxnExprConstraints()`             Restores the constraints on all flux expressions to the values contained in the fluxome. |
| `void` | `restoreRxnExprConstraints(java.util.Collection<? extends MathExpr<java.lang.String>> rxnNameExps)`             Restores the constraints on the provided expressions to the values contained in the fluxome. |
| `InitialPointComputer.Results` | `run()`             Computes an initial point, or flux vector, in the flux space of the fluxome used by this computer. |
| `void` | `setFluxConstraint(java.lang.String reactionName, Interval c)`             Changes the flux constraint of the reaction with the provided name. |
| `void` | `setFluxConstraints(java.util.Map<java.lang.String,Interval> fluxConstraints)`             Changes the flux constraints on more than one reaction. |
| `void` | `setPIntercept(double pIntercept)`             Sets the value of the positioning variables at the edges of the convex polytope. |
| `void` | `setRxnExprConstraint(MathExpr<java.lang.String> e, Interval c)`             Changes the constraint on the provided mathematical expression of fluxes. |
| `void` | `setRxnExprConstraints(java.util.Map<? extends MathExpr<java.lang.String>,Interval> constraints)`             Changes the constraints on more than one mathematical expression of fluxes. |

**Methods inherited from class java.lang.Object**

`clone, equals, finalize, getClass, hashCode, notify, notifyAll, toString, wait, wait, wait`

**Constructor Detail**

### InitialPointComputer

```
public InitialPointComputer(FbaOptComputer computer)
```

:   Constructs a new initial point, or flux vector, computer.

    **Parameters:**: `computer` - the computer with which optimizations will be performed.


**Method Detail**

### getFluxome

```
public Fluxome getFluxome()
```

:   Returns the fluxome used by this computer.

    :   **Specified by:**: `getFluxome` in interface `FluxCvComputer`
    :   **Returns:**: the fluxome used by this computer.

---


### getPIntercept

```
public double getPIntercept()
```

:   Returns the value of each positioning variable at the edges
    of the convex polytope.

    :   **Returns:**: the value of each positioning variable at the edges of the convex body.

---


### setPIntercept

```
public void setPIntercept(double pIntercept)
```

:   Sets the value of the positioning variables at the edges of the
    convex polytope. With a typical barrier function, this value is
    infinite, but with this linear "barrier function", it cannot be.

    :   **Parameters:**: `pIntercept` - a finite value > 10

---


### run

```
public InitialPointComputer.Results run()
```

:   Computes an initial point, or flux vector, in the flux space of the
    fluxome used by this computer.

    :   **Returns:**: the results of computing an initial point in the flux space of the fluxome used by this computer.

---


### setFluxConstraint

```
public void setFluxConstraint(java.lang.String reactionName,
                              Interval c)
```

:   Changes the flux constraint of the reaction with the provided name.

    :   **Specified by:**: `setFluxConstraint` in interface `FluxCvComputer`
    :   **Parameters:**: `reactionName` - the name of the reaction whose flux constraint is to be changed.: `c` - the new constraint.

---


### setFluxConstraints

```
public void setFluxConstraints(java.util.Map<java.lang.String,Interval> fluxConstraints)
```

:   Changes the flux constraints on more than one reaction.

    :   **Specified by:**: `setFluxConstraints` in interface `FluxCvComputer`
    :   **Parameters:**: `fluxConstraints` - reaction names mapped to their respective flux constraints.

---


### setRxnExprConstraint

```
public void setRxnExprConstraint(MathExpr<java.lang.String> e,
                                 Interval c)
```

:   Changes the constraint on the provided mathematical expression
    of fluxes.

    :   **Specified by:**: `setRxnExprConstraint` in interface `FluxCvComputer`
    :   **Parameters:**: `e` - a matheamtical expression of reaction names.: `c` - the new constraint.

---


### setRxnExprConstraints

```
public void setRxnExprConstraints(java.util.Map<? extends MathExpr<java.lang.String>,Interval> constraints)
```

:   Changes the constraints on more than one mathematical expression
    of fluxes.

    :   **Specified by:**: `setRxnExprConstraints` in interface `FluxCvComputer`
    :   **Parameters:**: `constraints` - mathematical expressions of reaction names mapped to their new respective constraints.

---


### restoreFluxConstraint

```
public void restoreFluxConstraint(java.lang.String reactionName)
```

:   Restores the flux constraint for the reaction with the provided
    name to the value contained in the fluxome.

    :   **Specified by:**: `restoreFluxConstraint` in interface `FluxCvComputer`
    :   **Parameters:**: `reactionName` - the name of the reaction whose flux constraint is to be restored.

---


### restoreFluxConstraints

```
public void restoreFluxConstraints(java.util.Collection<java.lang.String> reactionNames)
```

:   Restores the flux constraints for the provided reaction names to the
    values contained in the fluxome.

    :   **Specified by:**: `restoreFluxConstraints` in interface `FluxCvComputer`
    :   **Parameters:**: `reactionNames` - the reaction names whose flux constraints are to be restored.

---


### restoreFluxConstraints

```
public void restoreFluxConstraints()
```

:   Restores the flux constraints for all reactions in the fluxome to those
    values contained in the fluxome.

    :   **Specified by:**: `restoreFluxConstraints` in interface `FluxCvComputer`

---


### restoreRxnExprConstraint

```
public void restoreRxnExprConstraint(MathExpr<java.lang.String> expr)
```

:   Restores the constraint on the provided flux expression to the value
    contained in the fluxome.

    :   **Specified by:**: `restoreRxnExprConstraint` in interface `FluxCvComputer`
    :   **Parameters:**: `expr` - the expression of reaction names whose contraint is to be restored.

---


### restoreRxnExprConstraints

```
public void restoreRxnExprConstraints(java.util.Collection<? extends MathExpr<java.lang.String>> rxnNameExps)
```

:   Restores the constraints on the provided expressions to the values
    contained in the fluxome.

    :   **Specified by:**: `restoreRxnExprConstraints` in interface `FluxCvComputer`
    :   **Parameters:**: `rxnNameExps` - the reaction name expressions whose contraints are to be restored.

---


### restoreRxnExprConstraints

```
public void restoreRxnExprConstraints()
```

:   Restores the constraints on all flux expressions to the values
    contained in the fluxome.

    :   **Specified by:**: `restoreRxnExprConstraints` in interface `FluxCvComputer`

---


### getRxnExprConstraints

```
public java.util.Map<MathExpr<java.lang.String>,Interval> getRxnExprConstraints()
```

:   Returns all current flux expression constraints.

    :   **Specified by:**: `getRxnExprConstraints` in interface `FluxCvComputer`
    :   **Returns:**: mathematical expressions of reaction names mapped to their respective constraint.

---


### getFluxConstraints

```
public java.util.Map<java.lang.String,Interval> getFluxConstraints()
```

:   Returns all current flux constraints.

    :   **Specified by:**: `getFluxConstraints` in interface `FluxCvComputer`
    :   **Returns:**: reaction names mapped to their respective constraint.

---


### getRxnExprConstraint

```
public Interval getRxnExprConstraint(MathExpr<java.lang.String> rxnNameExp)
```

:   Returns the current constraint on the provided expression.

    :   **Specified by:**: `getRxnExprConstraint` in interface `FluxCvComputer`
    :   **Parameters:**: `rxnNameExp` - the mathematical expression of reaction names whose constraint is to be returned. **Returns:**: the current constraint on the provided expression.

---


### getFluxConstraint

```
public Interval getFluxConstraint(java.lang.String rxnName)
```

:   Returns the current flux constraint on the reaction with the provided
    name.

    :   **Specified by:**: `getFluxConstraint` in interface `FluxCvComputer`
    :   **Parameters:**: `rxnName` - the name of the reaction whose flux constraint is to be returned. **Returns:**: the current flux constraint on the reaction with the provided name.


---


|  |  |  |
| --- | --- | --- |
| **Overview** \| **Package** \| **Class** \| **Use** \| **Tree** \| **Deprecated** \| **Index** \| **Help** | |  |
| **PREV CLASS**   **NEXT CLASS** | **FRAMES**    **NO FRAMES**     **All Classes** |  |
| SUMMARY: NESTED \| FIELD \| CONSTR \| METHOD | DETAIL: FIELD \| CONSTR \| METHOD |  |


---
